# Impact of Physiotherapy in the Treatment of Pain in Cervical Dystonia

**DOI:** 10.5334/tohm.867

**Published:** 2024-03-06

**Authors:** Clemens Jacksch, Sebastian Loens, Joerg Mueller, Vera Tadic, Tobias Bäumer, Kirsten E. Zeuner

**Affiliations:** 1 Department of Neurology, Christian Albrechts-University of Kiel, Kiel, Germany; 2 Institute of Systems Motor Science, CBBM, University of Lübeck, Lübeck, Germany; 3 Centre of rare diseases, University Hospital Lübeck, Lübeck, Germany; 4 Department of Neurology, Vivantes Hospital Spandau, Berlin, Germany; 5 Department of Neurology, University Hospital Lübeck, Lübeck, Germany

**Keywords:** cervical dystonia, physiotherapy, pain, botulinum neurotoxin, quality of life

## Abstract

**Background:**

Cervical dystonia (CD) is the most common form of focal dystonia in adults. Studies show that physiotherapy (PT) in combination with BoNT has an effect on pain in cervical dystonia. We intended to test this hypothesis in a real-world setting to answer the question of whether pain is a good target symptom for prescribing PT. We also aimed to assess which form of PT is most appropriate for the treatment of pain.

**Methods:**

Study design: cross-sectional survey-based study of 91 patients with a confirmed diagnosis of cervical dystonia. The survey consisted of a questionnaire on type, frequency and content of physiotherapy, an assessment of quality of life with the Craniocervical Dystonia Questionnaire 24 (CDQ 24) and subjective pain scores.

**Results:**

53.8% of patients received physiotherapy, mostly a mixture of exercises to either correct the abnormal posture or to reduce the muscle tone. Additional therapies included stress-reducing exercises (14.3%), psychotherapy (9.9%) and EMG biofeedback (2.2%). Patients who received PT showed a non-significant tendency towards higher pain scores. The severity of dystonia-associated pain was significantly associated with the patients’ quality of life (F (1,54) = 22.9, adjusted R^2^ = 0.286, p < 0.001).

**Discussion:**

Pain is a frequent problem in patients with CD and severely affects quality of life. Physiotherapy could therefore be a valuable treatment option for patients with CD and pain.

**Highlights:**

Our uncontrolled study illustrates the high frequency of physiotherapy in addition to BoNT treatment in a real-life cohort of patients with cervical dystonia. We were able to show that PT reduces patients’ perceived pain in a patient reported outcome measure. This highlights the importance of PT in reducing CD-related pain, which considerably impairs quality of life.

## Introduction

Idiopathic cervical dystonia (CD) is the most common form of focal dystonia in adults. The prevalence of CD has been estimated between 28–183 cases/million with a 2:1 ratio in favor of the female gender [1]. CD is characterized by sustained or intermittent contractions of the cervical muscles that generate abnormal movements and postures [2]. More than half of the patients also present with concomitant dystonic head tremor [3]. In addition to motor impairments, several non-motor symptoms are associated with CD with pain being the most common in up to 70 % of patients [4]. The prevalence of dystonia associated pain was reported to affect 67%–75% of patients. In a recent metanalysis including 678 patients, pain was mild in 36 %, moderate in 42 % and severe in 21 % and improved significantly after IncobotulinumtoxinA treatment [4]. Other non-motor symptoms include depression, anxiety, sensory symptoms, impairment in sleep and fatigue [5,6].

First line treatment is the repeated injection of botulinum neurotoxin (BoNT) [7]. Also, deep brain stimulation (DBS) of the globus pallidus internus (GPI) is a sustained effective therapeutic option for more severe cases [8,9]. Pharmacological treatment with various oral drugs (predominantly anticholinergics such as trihexyphenidyl) is of limited efficacy [10]. Additional therapies, include miscellaneous forms of physiotherapy (PT), as well as stress-reducing techniques, electrotherapy and psychotherapy [11]. Since there is a lack of high-quality studies, two independent systematic reviews addressed the effect of PT in addition to BoNT treatment. The first one from 2014 left no clear recommendation for PT [12], while the second review from 2022 concluded that additional PT appeared to be useful [13]. The problem is that previous studies used a variety of technical approaches [13]. Not only the duration and frequency of PT sessions varied, but also the type of exercises [13]. The best-known specific exercise program (specialized physiotherapy) was described by Bleton. His program included not only stretching, elongation, and strengthening of the dystonic muscles, but also a training of the antagonists of those muscles that cause the involuntary dystonic movements [14]. Having said that, it appears that in regular physiotherapy similar techniques typically are applied by experienced physiotherapists anyway, so that additional benefits of a specialized versus a regular physiotherapy training program is a matter of debate [12,15,16].

The aim of our cross-sectional study was to determine the prevalence and structure of PT in a real-life setting in patients with CD. Furthermore, we investigated the subjective improvement of CD-associated pain by PT as reported by the patients.

## Methods

### Definition of the study cohort

For this cross-sectional study a total of 91 patients were recruited from the outpatient clinics for BoNT therapy at Kiel University Hospital and Lübeck University Hospital (Kiel n = 48, Lübeck n = 43). The study was approved by the local ethics committees of the universities of Kiel and Lübeck. All patients gave a written informed consent to participate.

The following inclusion criteria were applied:

- Diagnosis of idiopathic cervical dystonia or segmental dystonia with cervical predominance confirmed by a movement disorder expert.
- Patients must have been under BoNT treatment for at least 12 months, to ensure that PT and not an increase in botulinum toxin dosage improved subjective pain.

We excluded generalized and symptomatic forms of dystonia.

### Questionnaire based data collection

Patients reported their assessments either directly during their appointment at our BoNT outpatient clinic or during the course of the following treatment cycle. The part intended for the physiotherapists was then completed during the patient’s next physiotherapy session. Our patients and their treating physiotherapists were asked to indicate the focus and most frequently applied exercises of the last physiotherapy sessions when answering the questions. The questionnaire consisted of the following parts:

1. Collection of patient- and disease-specific information (age, sex, disease duration, medications, BoNT dosage). Patients were asked whether they were currently receiving physiotherapy.
2. Subjective pain scales a) Numeric Rating Scale (NRS), which graduates the neck pain on a scale from 0 to 10 and b) the patients’ subjective improvement/deterioration of neck pain after receiving PT.
3. Craniocervical Dystonia Questionnaire 24 (CDQ-24, German version [17]) recommended by the Movement disorder society (MDS).
4. One part was to be completed by the treating physiotherapists. For this purpose, our patients presented the questionnaire to their therapists during a PT session.

The physiotherapists were asked to fill in the following information:

- Frequency and duration of their therapy sessions
- What type of PT they used:
  - PT with a specific focus on dystonia-related symptoms
  - General (non-specific) PT that could also focus on other parts of the body rather than the head and neck (e.g. arms, legs, lower back)
- If the physiotherapists indicated that they used PT with a specific focus on dystonia-related symptoms, they had to choose whether they used:
  - PT techniques for postural correction and/or
  - Exercises to reduce increased muscle tone (with the following options to tick: passive mobilization, massage, relaxation)

The CDQ-24, a CD-specific assessment of quality of life with a special focus on CD-related problems in daily living [17], was obtained as a patient self-rating. It consists of 24 single items within five domains: *stigma* (question 7, 8, 9, 10, 18 and 22); *emotional well-being* (question 11, 12, 13, 14 and 15); *pain* (question 4, 5 and 21); *activities of daily living* (question 1, 2, 3, 6, 19 and 20) and *social/family life* (question 16, 17, 23 and 24). Each item ranks between 0 and 4 points, a higher score means a higher disease severity. The maximum score is 100 points, with 0 being the best and 100 being the worst possible CD-related Quality of Life [18].

We collected the rating of the TWSTRS severity score [19] and the details on the last BoNT treatment from the electronic medical records. The following BoNT preparations were used: OnabotulinumtoxinA (Botox®, Allergan, AbbVie company, Ireland), AbobotulinumtoxinA (Dysport®, Ipsen Pharma, France) and IncobotulinumtoxinA (Xeomin®, Merz Pharmaceuticals, Germany). To ensure comparability of the applied BoNT preparations, we used the following formula to calculate the equivalent dose for AbobotulinumtoxinA: 1 U OnaA or IncoA = 2.5 U AboA. This ratio showed similar efficacy and side effects according to a systematic literature review [20]. The TWSTRS was assessed in an unblinded manner prior to the injection of BoNT during the patients’ visit to our BoNT outpatient clinic.

### Statistical Analysis

Parametric and nonparametric t-tests, Chi-Square-test as well as linear and multiple linear regression with backwards stepwise data entry were used for data analyses.

Statistical significance was assumed at a p-value of less than 0.05. The statistical analysis was performed with “JASP” (version 0.14, University of Amsterdam, Amsterdam, The Netherlands).

## Results

The mean age of the study cohort was 62.4 (SD ± 11.2) years, and 76.9% of patients were female. The mean disease duration was 15.5 (SD ± 11.3) years (Table 1). Concomitant medications included beta-blockers (29.7%), antidepressants (14.3%), pregabalin and gabapentin (7.7%), benzodiazepines (4.4%), trihexyphenidyl (4.4%), levodopa (3.3%), tizanidine (3.3%), as well as pridinol, tiapride, dronabinol and primidone (each taken by one patient).

**Table 1:** Characteristics of the study cohort (n = 91).


Sex	

female:	70 (76.9%)

male:	21 (23.1%)

Age	62.4 (SD ± 11.2) years

	Maximum: 84 years

	Minimum: 32 years

Disease duration	15.5 (SD ± 11.3) years

	Maximum: 52 years

	Minimum: 1 year

BoNT treatment time	10.2 (SD ± 7.7) years

	Maximum: 32 years

	Minimum: 1 year

BoNT mean absolute dose* (Max./Min.)	IncoA: 218.6 U (420/90)

	AboA: 1405.2 U (2400/350)

	OnaA: 180.5 U (300/50)


* Applied conversion of the BoNT equivalent dose: 1 U OnaA or IncoA = 2,5 U AboA [19]. * SD: standard deviation.

### 1. Physiotherapy treatment

In our cohort, 53.8% of patients reported concomitant PT. According to the information of the treating physiotherapists, 59.2 % of patients received PT with a special focus on dystonia related symptoms, while in 40.8% of patients a general PT was applied.

In those cases that were marked as “dystonia specific PT” we further divided the techniques between exercises for posture correction and exercises to reduce increased muscle tone following the information provided by the physiotherapists.

**Figure 1:**
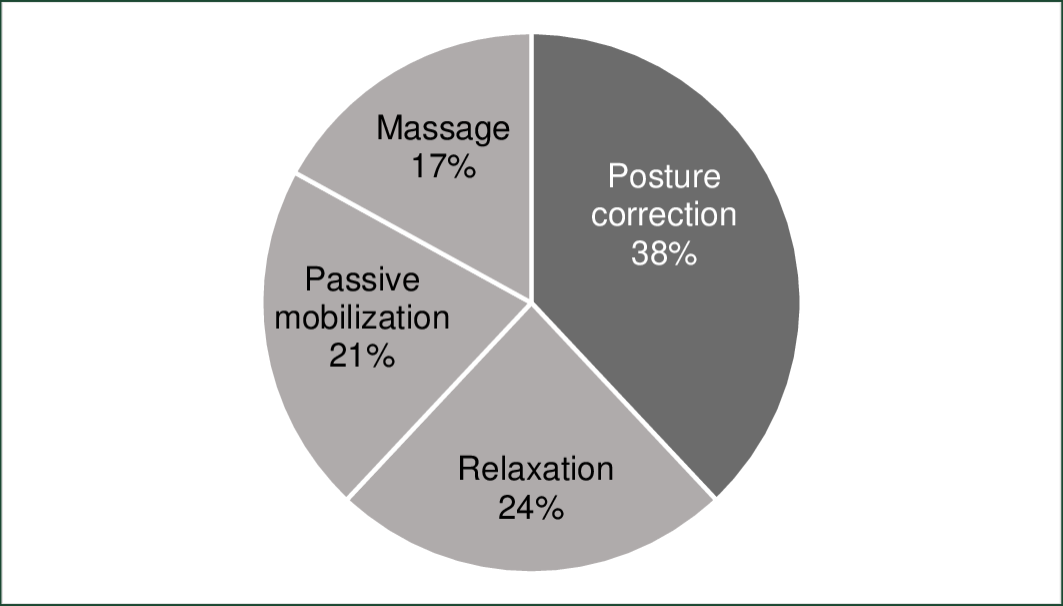
Distribution of the different techniques used in the study cohort (dark grey: exercises for posture correction, light grey: exercises for reducing muscle tone).

The distribution of the different techniques used in the study cohort is shown in Figure 1.

Patients were treated an average of 1.8 times per week (max. 5 times, min. once) with an average duration of 1.2 hours per week. Besides PT, patients used stress-reducing exercises (e.g. yoga, autogenic training, progressive muscle relaxation) (14.3%), psychotherapy (9.9%) and EMG biofeedback (2.2%). In addition, a few patients received electrotherapy, acupuncture, occupational therapy, osteopathy, and water gymnastics.

We used multiple univariate regression analyses to identify factors that may predict the utilization of PT. When entered separately into the model, age, sex, disease duration, BoNT dose, TWSTRS severity score, CDQ 24 total score, NRS and the pain subitem of the CDQ 24 showed no significant association with PT usage.

### 2. Relationship between additional physiotherapy, motor scores and pain

#### Motor scores

We collected the TWSTRS severity score in 59 of 91 patients. The TWSTRS mean score was 17.5 (± 3.7). There was no significant difference in TWSTRS severity score between patients with (17.7 SD ± 3.7) or without (17.1 SD ± 3.8) concomitant PT (p = 0.567). There were also no differences in TWSTRS between patients receiving dystonia-specific PT and patients receiving general PT (p = 0.828).

#### Pain

**Figure 2:**
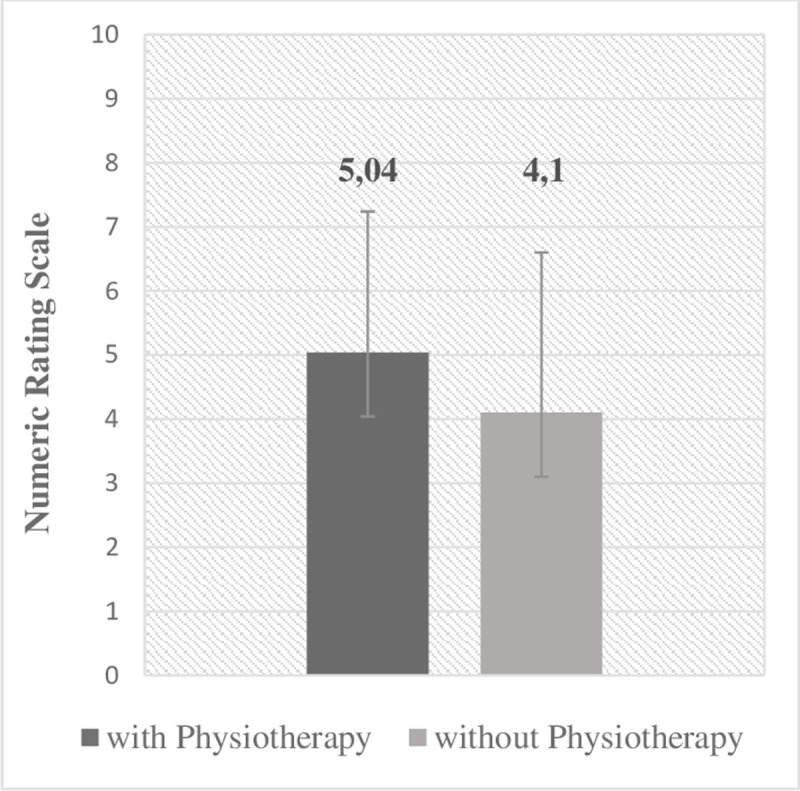
Score of the Numeric Rating Scale in patients with and without physiotherapy.

Overall, 94.5% of patients reported dystonia-associated pain. Only 5 patients reported no pain. The overall mean pain intensity on the numeric rating scale (NRS) was 4.6 (SD ± 2.4). Patients with accompanying PT tended to have higher pain scores (NRS 5.04 SD ± 2.2 vs. 4.1 SD ± 2.5, p = 0.065), as illustrated in Figure 2.

In their subjective assessment, all patients indicated a noticeable benefit in pain after receiving physiotherapy, with an average improvement of 51% (SD ± 23%). Only two patients reported an alternating effect on their pain with sometimes a 50% benefit or a 50% worsening afterwards. The positive benefit on pain after PT did not differ between patients receiving dystonia-specific PT and patients receiving a more general PT (p = 0.468).

### 3. CDQ 24 quality of life questionnaire and relationship with concomitant physiotherapy

The mean CDQ 24 total score was 30 (SD ± 18.1) points, and for the single subitems: *stigma* 8.6 (SD ± 6.5), *emotional well-being* 6.2 (SD ± 4.5), *pain* 4.4 (SD ± 3.2), *activities of daily living* 8.3 (SD ± 5.2), and *social and family life* 2.5 (SD ± 2.9) points.

Comparing patients with and without PT, the mean CDQ 24 total score showed a trend for higher scores in patients with PT indicating lower QoL (33.4 ± SD 18.6 vs. 26.1 ± SD 16.9, p = 0.068).

There was no difference in CDQ 24 total score between patients with a PT specifically focused on dystonia-related symptoms and patients with a more general PT (p = 0.166).

Exploratory analysis of the CDQ 24 subitems revealed that patients with PT reported higher scores for the “activities of daily living” subitem (with PT 9.8 ± 5.3; without PT 6.6 ± 4.6; p = 0.004, Bonferroni corrected for multiple comparisons).

### 4. Influence of patient- and disease-specific markers on the CDQ 24

We performed multiple linear regression with backwards stepwise data entry to select the best model to predict the CDQ24 total score. The CDQ24 total score was the dependent variable, whereas age, disease duration, BoNT dose, TWSTRS severity score, and severity of dystonia-associated pain as measured with the NRS served as covariates. All assumptions of collinearity, normality, linearity and homoscedasticity were not violated. Variables with less than a significant contribution were removed. The analyses revealed that among the analyzed items dystonia-associated pain was the only factor that significantly predicted the level of the CDQ24 total score and thus significantly influenced quality of life (F (1,54) = 22.9, adjusted R^2^ = 0.286, p < 0.001). The remaining covariates had no significant predictive power regarding the CDQ24 total score.

The results for the use of PT, the motor, pain and CDQ24 scores are summarized in Table 2.

**Table 2:** Presentation of the utilization of physiotherapy, motor scores, pain scores and CDQ24 scores of the study cohort (n = 91).


UTILIZATION OF PHYSIOTHERAPY	

yes	49 (53.8%)

Dystonia-specific PT	29 (31.9%)

General PT	20 (21.9%)

no	42 (46.2%)

**TWSTRS severity score**	

(n = 59)	17.5 (SD ± 3.7)

with PT	17.7 (SD ± 3.7)

without PT	17.1 (SD ± 3.8)

**Pain Scores**	

Numeric Rating Scale	4.6 (SD ± 2.4)

with PT	5.04 (SD ± 2.2)

without PT	4.1 (SD ± 2.5)

CDQ 24 Subitem Pain	4.4 (SD ± 3.2)

with PT	4.9 (SD ± 3.0)

without PT	3.8 (SD ± 3.3)

**CDQ 24 subscores**	

Mean Score	30 (SD ± 18.1)

with PT	33.4 ± SD 18.6

without PT	26.1 ± SD 16.9

Stigma	8.6 (SD ± 6.5)

with PT	8.9 (SD ± 6.5)

without PT	8.1 (SD ± 6.6)

Emotional well-being	6.2 (SD ± 4.5)

with PT	6.7 (SD ± 5.2)

without PT	5.6 (SD ± 3.6)

Activities of daily living	8.3 (SD ± 5.2)

with PT	9.8 (SD ± 5.3)

without PT	6.6 (SD ± 4.6)

Social and family life	2.5 (SD ± 2.9)

with PT	2.9 (SD ± 3.1)

without PT	1.9 (SD ± 2.5)


## Discussion

The aim of our cross-sectional study was to determine the impact of physiotherapy on pain in patients with cervical dystonia. Furthermore, we were interested in the type of treatment patients received in a real-life setting as documented by their physiotherapists.

In our cohort 53.8 % of the patients received concomitant PT. According to the documentation of the treating physiotherapists, 60.4 % of patients obtained PT with a focus on dystonia specific symptoms, while 39.6 % took advantage of general PT with no special focus on CD. There was a wide range of techniques physiotherapists used. They included PT exercises for posture correction as well as exercises to decrease muscle tone such as massage, relaxation and passive mobilization in a balanced distribution. We obtained the following major results:

1. Most of our patients (94.5%) reported dystonia-associated pain as captured with the NRS and CDQ-24 pain subitem.
2. Dystonia associated pain had a significant negative influence on quality of life (R^2^ = 0.286, p < 0.001).
3. In a real-world setting, patients who received PT tended to have higher levels of dystonia-associated pain than patients who did not receive therapy (p = 0.065).
4. Almost all patients (n = 89/91) indicated a noticeable benefit in pain with an average improvement of 51% (SD ± 23%) when receiving PT.
5. Most patients received a mixture of different treatment forms such as exercises to decrease the muscle tone or to correct head and neck posture.

In line with the literature [4,5], pain had been identified as a major complaint in our patients. Our results showed that among all factors in the analysis only dystonia-associated pain had a significant negative impact on quality of life in patients with CD and nearly all patients reported a subjective improvement in pain of at least 50% when performing PT. This corresponds to findings in previous studies [12,13]. PT may also have an additional positive effect on pain in combination with BoNT injections as it was the case in our study. For example, Tassorelli et al. demonstrated longer-lasting clinical benefit of BoNT injection and lower follow-up dose for the next injection, as well as improvement in activities of daily living and subjective pain in combination with PT [21]. Secondly, a PT rehabilitation protocol in combination with BoNT resulted in a beneficial effect on pain [22]. Finally, in a one-year study with 96 CD patients, pain significantly improved with PT, regardless whether it was specialized or not [23]. Although larger randomized controlled PT studies are missing, it is likely that patients benefit with respect to their CD associated pain.

We found that in addition to higher pain levels, patients with PT also scored higher on the CDQ 24 subitem “activities of daily living” (9.8 ± 5.3 vs. 6.6 ± 4.6; p = 0.004), indicating a more generalized impairment in QoL. In contrast to our results, Klingelhöfer et al [24] described higher disability and worse motor symptoms as measured in the TWSTRS in those patients with limitations in the ADL. In our study dystonia-associated pain was the only factor that significantly predicted the level of the CDQ24 total score and thus significantly influenced quality of life. Therefore, it is likely that pain also influenced the activity of daily living, which is in line with studies showing that chronic pain has a multi-dimensional impact on QoL [24]. Pain is known to affect patient’s emotional well-being, is correlated with depression [24] and patients’ ability to participate in activities or to work [4]. Adjunctive PT might alleviate pain and thereby improve quality of life as proposed by Loudovici-Krug et al in their systematic review [13].

Since there is an ongoing academic discussion, whether the PT techniques applied for CD should specifically address the patients’ dystonic posturing or not, we were interested to know what kind of PT is applied in the daily routine. The most well-known specialized PT method is the personalized intensive physiotherapy program described by Bleton [14], who combined relaxation of overactive and strengthening of underactive muscles with posture correction and enhancing the awareness of the body’s midline position. However, a randomized PT trial compared Bleton’s technique to standard PT and reported sustained improvement of motor scores in both groups without any differences [15]. A more recent study compared the impact of a specialized versus regular physiotherapy in addition to BoNT in 72 patients. Again, there were no differences between groups in terms of the TWSTRS disability scale and pain as measured with the NRS and the pain subscore of the TWSTRS. However, patients in the group with specialized PT perceived higher subjective effects in the Clinical Global Impression-Improvement Scale and in overall health perception as captured with a subitem of the SF-36 after a treatment period of 12 months [16,23]. The question that arises from those studies is, whether there is a “specific” physiotherapy needed or perhaps more general and specific parts are combined anyway in a PT program.

The classification of the PT methods applied in our study as specific or unspecific was based exclusively on the judgment of the treating physiotherapist and did not follow the training suggested by Bleton [14]. We found no differences between physiotherapy with a specific focus on CD and a general physiotherapy in terms of dystonia severity as measured by the TWSTRS, as well as pain and patients’ quality of life. Corresponding to the findings and conclusions of Counsell et al. [15] it is likely that experienced physiotherapists employ dystonia-specific techniques routinely when required, independently of Bleton’s personalized physiotherapy program. This has been confirmed by a most recent meta-analysis that included 14 studies with 414 CD patients. There was a benefit for CD patients who received the combination of BoNT and PT and had good benefit especially in regard to pain, but the authors gave no clear recommendation for a specific type of PT [25].

## Conclusion

Our study illustrates the high frequency of physiotherapy and other adjunctive therapies in addition to BoNT treatment in a real-life cohort of patients with cervical dystonia. We were able to highlight the importance of PT to reduce pain associated with CD, which significantly affects quality of life. Although, PT is not an evidence-based treatment option in CD our results suggest that PT should be prescribed on a regular basis in addition to BoNT.

## Strengths and limitations

The strength of our study is the comprehensive and differentiated investigation of applied additional therapies in a large number of CD patients under real-world conditions.

Limitations arise from the uncontrolled nature of the study and the different intervals between physiotherapy sessions and BoNT injections in the individual patients.

## Financial Disclosures

**CJ** declares that there are no additional disclosures to report.

**SL** received honoraria from IPSEN Pharma and Merz Pharmaceuticals.

**JM** received speakers honoraria from Esteve and Merz.

**VT** declares that there are no additional disclosures to report.

**TB** is an employee of the University Hospital Schleswig Holstein.

He received funding from the German Research Foundation (DFG, BA 6375/2-1).

He received speaker and consultant fees from Pelzerhaken Children’s Centre, Allergan/Abbvie, Ipsen Pharma and Merz Therapeutics.

He has received research funding from: Allergan/Abbvie, Ipsen Pharma and Merz Therapeutics. He was supported with exhibition ultrasound equipment on loan from Cannon and ESAOTE.

**KEZ** has received research support from the Christa and Hans-Peter Thomsen Foundation, the German Research Foundation (DFG 5919/4-1) and from Strathmann GmbH & Co. KG.

She reports speaker’s honoraria from Bayer Vital GmbH, BIAL, Alexion, AbbVie and Merz outside the submitted work.

She has served as a consultant and received fees from Merz, Ipsen, Alexion and the German Federal Institute for Drugs and Medical Devices (BfArM).

## Additional Files

The additional files for this article can be found as follows:

10.5334/tohm.867.s1 Supplementary file 1. Questionnaire 1 and 2

10.5334/tohm.867.s2 Supplementary file 2. Fragebogen 1 und 2

## Abbreviations

CD: Cervical dystonia; PT: Physiotherapy; BoNT: Botulinum neurotoxin; CDQ24: Craniocervical Dystonia Questionnaire 24; DBS: Deep brain stimulation; GPI: Globus pallidus internus; NRS: Numeric Rating Scale.
