# Supplementary material for: Impact of Physiotherapy in the Treatment of Pain in Cervical Dystonia
Source: Tremor Other Hyperkinet Mov (N Y). 2024 Mar 6;14:11. doi: 10.5334/tohm.867 (PMC10921958; doi:10.5334/tohm.867)
Supplement: Supplementary file 1. — Questionnaire 1 and 2 [file tohm-14-1-867-s1.pdf]

Questionnaire 1

General information:

Age: _______ Gender: ☐ male ☐ female

Onset of illness: ______ Therapy with botulinum toxin since: ______ Last botulinum toxin injection on: ________

Medication: ____

Accompanying therapies to botulinum toxin: (please check each ☐ mark with a cross if applicable)

1) **No concomitant therapy** ☐

2) **Physiotherapy specially designed for dystonia** ☐

For example:
Exercises focusing on the correction of dystonic malposition; stretching of the dystonic muscles with subsequent passive movement; so-called "Bleton" method: strengthening of the muscles contrary to the (dystonic) direction of pull

Description of the method used (physiotherapist part):

………

………

………

3) **„General" physiotherapy and not specifically designed for dystonia** ☐

Description of the method used (physiotherapist part):

a) Posture correction O (____ % of therapy time)
b) Relaxation O (____ % of therapy time)
c) Muscle stretching O (____ % of therapy time)
d) Passive mobilization O (____ % of therapy time)
e) Massage O (____ % of therapy time)

4) **Other therapies:** ☐

Relaxation exercises (e.g. progressive muscle relaxation) O
Psychotherapy (e.g. cognitive behavioral therapy) O
Electrotherapy (e.g. transcutaneous electrical stimulation TENS) O
EMG biofeedback training O
Other: ________ O

Frequency (e.g. how often per week for how many hours):

___ per week; each ____ hours

UKSH Wissen schafft Gesundheit

Questionnaire 2

How often did the following statements apply to you <u>within the last 14 days</u> due to the dystonia disorder?

Please check one answer option and answer each question if possible.

1) Did you have problems reading or watching TV?
never ○ occasionally ○ sometimes ○ often ○ always ○

2) Did you have difficulties doing activities that you wanted to do? (e.g. leisure, hobby)?
never ○ occasionally ○ sometimes ○ often ○ always ○

3) Did you have difficulty controlling the symptoms of your illness when you were stressed or upset?
never ○ occasionally ○ sometimes ○ often ○ always ○

4) Have you suffered from pain/burning in the face, head or neck area?
never ○ occasionally ○ sometimes ○ often ○ always ○

5) Did pain or a pulling sensation prevent you from falling asleep?
never ○ occasionally ○ sometimes ○ often ○ always ○

6) Did you have difficulty performing fine tasks with your fingers (e.g. writing, threading)?
never ○ occasionally ○ sometimes ○ often ○ always ○

7) Did you avoid situations where many people were present (e.g. social events)?
never ○ occasionally ○ sometimes ○ often ○ always ○

8) Did you feel uncomfortable in public because of your illness?
never ○ occasionally ○ sometimes ○ often ○ always ○

9) Did you have the feeling that you had to hide your illness from others?
never ○ occasionally ○ sometimes ○ often ○ always ○

10) Have you thought about how other people react to you?
never ○ occasionally ○ sometimes ○ often ○ always ○

UKSH Wissen schafft Gesundheit

11) Have you been worried about your future?

never ○ occasionally ○ sometimes ○ often ○ always ○

12) Did you feel anxious?

never ○ occasionally ○ sometimes ○ often ○ always ○

13) Did you feel down or depressed?

never ○ occasionally ○ sometimes ○ often ○ always ○

14) Did you feel sad or on the verge of tears?

never ○ occasionally ○ sometimes ○ often ○ always ○

15) Were you angry or bitter?

never ○ occasionally ○ sometimes ○ often ○ always ○

16) Did your illness make you feel isolated or lonely?

never ○ occasionally ○ sometimes ○ often ○ always ○

17) Have you had problems with close friends or family as a result of your illness?

never ○ occasionally ○ sometimes ○ often ○ always ○

18) Did you feel insecure or tense when dealing with people you didn't know?

never ○ occasionally ○ sometimes ○ often ○ always ○

19) Did you have difficulties coping with the demands placed on you at work or at home?

Employed: yes ○ *no* ○

not at all ○ low ○ moderate ○ strong ○ very strong ○

20) Did you feel impaired in traffic?

not at all ○ low ○ moderate ○ strong ○ very strong ○

21) Did you feel affected by pain/burning in the face, head or neck area?

not at all ○ low ○ moderate ○ strong ○ very strong ○

22) Have you had the feeling of being less attractive on the outside?

not at all ○ low ○ moderate ○ strong ○ very strong ○

23) Has the dystonia disorder put a strain on your family life?

not at all ○ low ○ moderate ○ strong ○ very strong ○

24) Has the dystonia disorder affected your partner relationship?

Relationship: yes ○ *no* ○

not at all ○ low ○ moderate ○ strong ○ very strong ○

Pain scale:

How would you rate your pain in connection with cervical dystonia (e.g. accompanying head, neck, shoulder and back pain)? **please check the number**

0 (no pain) 10 (strongest imaginable pain)

0 1 2 3 4 5 6 7 8 9 10

In the case of concomitant therapy: How do you rate the pain after the therapy?

a) ____ % improvement O

b) ____ % deterioration O
