# Supplementary material for: Impact of Physiotherapy in the Treatment of Pain in Cervical Dystonia
Source: Tremor Other Hyperkinet Mov (N Y). 2024 Mar 6;14:11. doi: 10.5334/tohm.867 (PMC10921958; doi:10.5334/tohm.867)
Supplement: Supplementary file 2. — Fragebogen 1 und 2 [file tohm-14-1-867-s2.pdf]

FRAGEBOGEN 1

Allgemeine Angaben:

Alter: _______ Geschlecht: ☐ männlich ☐ weiblich

Krankheitsbeginn: ______ Therapie mit Botulinumtoxin seit: ______ Letzte Botulinumtoxin-Injektion am: ________

Medikamente: ____

Begleitende Therapien zu Botulinumtoxin: (bitte jeweils ☐ ankreuzen falls zutreffend)

1) **Keine begleitende Therapie** ☐

2) **Speziell auf die Dystonie ausgelegte Physiotherapie** ☐

Zum Beispiel:
Übungen mit Schwerpunkt auf der Korrektur der dystonen Fehlhaltung; Dehnung der dystonen Muskultatur mit anschließender passiver Bewegung; sogenannte „Bleton"-Methode: Stärkung der Muskeln konträr zur (dystonen) Zugrichtung

Beschreibung der angewandten Methode (Physiotherapeuten-Teil):

……….

……….

……….

3) **„Normale" und nicht speziell auf die Dystonie ausgelegte Physiotherapie** ☐

Beschreibung der angewandten Methode (Physiotherapeuten-Teil):

a) Korrektur der Körperhaltung O (____ % der Therapie-Zeit)
b) Relaxation O (____ % der Therapie-Zeit)
c) Muskeldehnung O (____ % der Therapie-Zeit)
d) passive Mobilisation O (____ % der Therapie-Zeit)
e) Massage O (____ % der Therapie-Zeit)

4) **Andere Therapien:** ☐

Entspannungsübungen (z.B. progressive Muskelrelaxation) O
Psychotherapie (z.B. kognitive Verhaltenstherapie) O
Elektrotherapie (z.B. Transkutane Elektrostimulation TENS) O
EMG-Biofeedbacktraining O
Andere: ________ O

Frequenz (z.B. wie oft pro Woche über wie viele Stunden):

___ pro Woche; jeweils ____ Stunde

Fragebogen 2

Wie häufig trafen bei Ihnen aufgrund der Dystonie-Erkrankung <u>innerhalb der letzten 14 Tage</u> die folgenden Angaben zu?

Bitte kreuzen Sie eine Antwortmöglichkeit an und beantworten nach Möglichkeit jede Frage.

1) Hatten Sie Probleme beim Lesen oder Fernsehen?
   nie ○ gelegentlich ○ manchmal ○ häufig ○ immer ○

2) Hatten Sie Schwierigkeiten mit der Ausübung von Aktivitäten, zu denen Sie Lust hatten? (z.B. Freizeit, Hobby)?
   nie ○ gelegentlich ○ manchmal ○ häufig ○ immer ○

3) Hatten Sie Schwierigkeiten, bei Stress oder Aufregung die Symptome ihrer Erkrankung zu kontrollieren?
   nie ○ gelegentlich ○ manchmal ○ häufig ○ immer ○

4) Haben Sie unter Schmerzen / Brennen im Gesicht, Kopf- oder Nackenbereich gelitten?
   nie ○ gelegentlich ○ manchmal ○ häufig ○ immer ○

5) Hinderten Sie Schmerzen oder ein Ziehen am Einschlafen?
   nie ○ gelegentlich ○ manchmal ○ häufig ○ immer ○

6) Hatten Sie Schwierigkeiten, feine Tätigkeiten mit den Fingern durchführen (z.B. Schreiben, Faden einfädeln)?
   nie ○ gelegentlich ○ manchmal ○ häufig ○ immer ○

7) Vermieden Sie Situationen, bei denen viele Menschen anwesend waren (z.B. gesellschaftliche Veranstaltungen)?
   nie ○ gelegentlich ○ manchmal ○ häufig ○ immer ○

8) Fühlten Sie sich in der Öffentlichkeit aufgrund Ihrer Erkrankung unwohl?
   nie ○ gelegentlich ○ manchmal ○ häufig ○ immer ○

9) Hatten Sie das Gefühl, Ihre Erkrankung vor Anderen verbergen zu müssen?
   nie ○ gelegentlich ○ manchmal ○ häufig ○ immer ○

10) Haben Sie sich Gedanken gemacht, wie andere Leute auf Sie reagieren?
   nie ○ gelegentlich ○ manchmal ○ häufig ○ immer ○

11) Haben Sie sich Sorgen über Ihre Zukunft gemacht?

nie ○ gelegentlich ○ manchmal ○ häufig ○ immer ○

12) Haben Sie sich ängstlich gefühlt?

nie ○ gelegentlich ○ manchmal ○ häufig ○ immer ○

13) Fühlten Sie sich niedergeschlagen oder deprimiert?

nie ○ gelegentlich ○ manchmal ○ häufig ○ immer ○

14) Fühlten Sie sich traurig oder den Tränen nahe?

nie ○ gelegentlich ○ manchmal ○ häufig ○ immer ○

15) Waren Sie verärgert oder verbittert?

nie ○ gelegentlich ○ manchmal ○ häufig ○ immer ○

16) Fühlten Sie sich durch Ihre Erkrankung isoliert oder einsam?

nie ○ gelegentlich ○ manchmal ○ häufig ○ immer ○

17) Hatten Sie durch Ihre Erkrankung Probleme im Umgang mit engen Freunden oder Ihrer Familie?

nie ○ gelegentlich ○ manchmal ○ häufig ○ immer ○

18) Fühlten Sie sich im Umgang mit Ihnen unbekannten Menschen unsicher oder angespannt?

nie ○ gelegentlich ○ manchmal ○ häufig ○ immer ○

19) Hatten Sie Schwierigkeiten, die an Sie gestellten Anforderungen in Beruf oder Haushalt zu bewältigen?

Berufstätig: Ja ○ *Nein* ○

gar nicht ○ gering ○ mäßig ○ stark ○ sehr stark ○

20) Fühlten Sie sich im Straßenverkehr beeinträchtigt?

gar nicht ○ gering ○ mäßig ○ stark ○ sehr stark ○

21) Fühlten Sie sich durch Schmerzen/ Brennen im Gesicht, Kopf- oder Nackenbereich beeinträchtigt?

gar nicht ○ gering ○ mäßig ○ stark ○ sehr stark ○

22) Haben Sie das Gefühl gehabt, äußerlich weniger attraktiv zu sein?

gar nicht ○ gering ○ mäßig ○ stark ○ sehr stark ○

23) Hat die Dystonie-Erkrankung Ihr Familienleben belastet?

gar nicht O gering O mäßig O stark O sehr stark O

24) Hat die Dystonie-Erkrankung Ihre Partnerbeziehung beeinträchtigt?

Partner: Ja O *Nein* O

gar nicht O gering O mäßig O stark O sehr stark O

Schmerzskala:

Wie bewerten Sie Ihre Schmerzen in Zusammenhang mit der zervikalen Dystonie (zum Beispiel begleitende Kopf-, Nacken-, Schulter- und Rückenschmerzen)? **bitte Ziffer ankreuzen**

0 (keine Schmerzen) 10 (stärkster vorstellbarer Schmerz)

0 1 2 3 4 5 6 7 8 9 10

Im Falle einer begleitenden Therapie: Wie bewerten Sie die Schmerzen nach der Therapie?

a) ____ % Verbesserung O

b) ____ % Verschlechterung O
